# Asymptomatic, Mild, and Severe Influenza A(H7N9) Virus Infection in Humans, Guangzhou, China

**DOI:** 10.3201/eid2009.140424

**Published:** 2014-09

**Authors:** Zongqiu Chen, Hui Liu, Jianyun Lu, Lei Luo, Kuibiao Li, Yufei Liu, Eric H.Y. Lau, Biao Di, Hui Wang, Zhicong Yang, Xincai Xiao

**Affiliations:** Guangzhou Center for Disease Control and Prevention, Guangzhou, China (Z. Chen, H. Liu, J. Lu, L. Luo, K. Li, Y. Liu, B. Di, H. Wang, Z. Yang, and X. Xiao);; University of Hong Kong, Hong Kong, China (E.H.Y. Lau)

**Keywords:** avian influenza, influenza, influenza A(H7N9), H7N9, surveillance, person-to-person transmission, asymptomatic infection, viruses, respiratory infections, human, China

Targeted surveillance for influenza A(H7N9) identified 24 cases of infection with this virus in Guangzhou, China, during April 1, 2013–March 7, 2014. The spectrum of illness ranged from severe pneumonia to asymptomatic infection. Epidemiologic findings for 2 family clusters of infection highlight the importance of rigorous close contact monitoring.

During February–May 2013, the initial outbreak of human infection with avian influenza A(H7N9) virus in China resulted in 133 cases (*1*). Influenza A(H7N9) virus reemerged in southern China in October 2013 and had caused 85 laboratory-confirmed cases of infection in Guangdong Province as of March 7, 2014. In response to the outbreak, targeted surveillance programs were established in April 2013 in Guangzhou, the provincial capital of Guangdong Province. Here, we report results of this surveillance program through early 2014.

## The Study

Since 2004, all clinical facilities in Guangzhou (population 13 million in 2013) have been required by the China National Health and Family Planning Commission to report any patient who meets the criteria of having pneumonia of unknown etiology (PUE): fever (>38°C), radiologic characteristics consistent with pneumonia, low-normal leukocyte count or low lymphocyte count in early-stage disease, and no alternative etiology (*2*). Upper or lower respiratory samples from these patients are collected for identification of possible causative pathogens. In response to the influenza A(H7N9) outbreak, PUE surveillance was enhanced in April 2013 by implementing laboratory testing specific for influenza A(H7N9) virus (*3*). Specimens are initially screened for influenza A and B viruses by real-time reverse transcription PCR (rRT-PCR); samples positive for influenza A are then subtyped as H1N1, H3N2, H5N1, or H7N9.

Surveillance for influenza-like illness (ILI) was initially conducted in 4 sentinel hospitals in Guangzhou and expanded to 19 hospitals in November 2013. Each hospital collects 10–20 convenience throat swab specimens weekly from ILI patients visiting the hospitals within 3 days of illness onset. The same laboratory screening protocols were adopted as for PUE surveillance.

Surveillance for influenza A(H7N9) virus was established in 24 live poultry markets (LPMs) in April 2013 and expanded to 42 LPMs in November 2013, covering all 12 districts in Guangzhou. From each LPM, 10–30 environmental samples are collected biweekly and tested by rRT-PCR. When human influenza A(H7N9) infection is confirmed, additional environmental sampling from epidemiologically linked LPMs is immediately launched to trace the possible source of infection. All poultry workers linked to influenza A(H7N9) virus–contaminated LPMs (i.e., LPMs with >1 virus-positive environmental samples identified) are placed under medical observation for 7 days. Throat swab specimens are collected within 24 hours for detection of influenza A(H7N9) infection and second swab specimens are collected if symptoms appear.

Close contacts of influenza A(H7N9) case-patients are defined as any family member who shares residence, social contacts who visit, and health care workers who provide medical services without effective personal protection (*4*) during the period from 1 day before illness onset to isolation (*5*). All close contacts are monitored for 7 days for any symptoms. Pared serum samples, tested by hemagglutinin inhibition assay (*6*), and throat swab specimens are collected to detect possible secondary cases.

**Figure 1:**
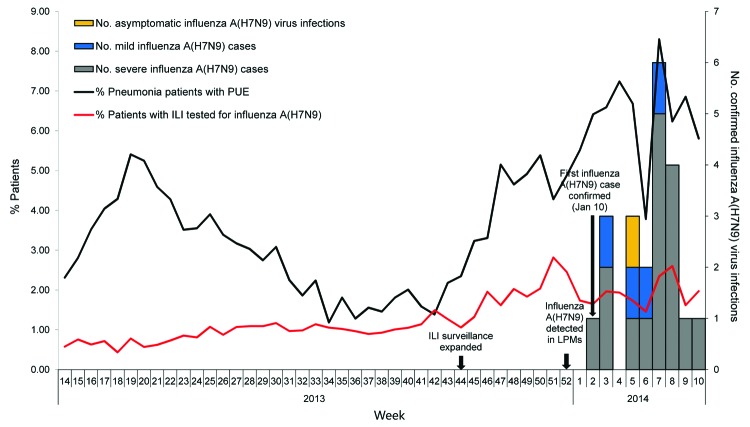
Weekly number of confirmed influenza A(H7N9) cases detected by real-time reverse transcription PCR, percentage of pneumonia patients with pneumonia of unknown etiology (PUE), and percentage of patients with influenza-like illness (ILI) tested for influenza A(H7N9), Guangzhou, China, April 1, 2013–March 7, 2014. For PUE and ILI surveillance in Guangzhou, laboratory testing for influenza A(H7N9) virus using real-time reverse transcription PCR was implemented in week 14 of 2013 (April 1, 2013). ILI surveillance was expanded to 19 sentinel hospitals in week 44 (November 2013), according to the requirements of the public health authority of Guangdong Province after 4 confirmed influenza A(H7N9) cases were reported in Guangdong.

From April 1, 2013, through March 7, 2014, a total of 47,937 patients with pneumonia were reported in Guangzhou (Table 1). Of these, 1,923 (4.0%) met PUE criteria, and respiratory specimens were collected and tested. An influenza A(H7N9) case in Guangzhou was confirmed on January 10, 2014 (Figure 1); since then, an additional 15 patients with PUE were confirmed as influenza A(H7N9) case-patients. All were adults; 11 (69%) were >60 years of age. Recent poultry exposure history was available for 14 (88%) patients (Table 2).

**Table 1:** Investigation of weekly reported number of patients with pneumonia, PUE, and ILI and confirmed cases of influenza A(H7N9) virus infection, Guangzhou, China, April 1, 2013–March 7, 2014*

Year, wk	PUE surveillance				ILI surveillance		
	No. patients with pneumonia	No. (%) patients with PUE	No. confirmed influenza A(H7N9) infections		No. patients with ILI	No. (%) samples tested	No. confirmed influenza A(H7N9) infections
2013							
14	951	22 (2.3)	0		8,089	47 (0.6)	0
15	996	28 (2.8)	0		8,555	65 (0.8)	0
16	1,021	36 (3.5)	0		8,698	55 (0.6)	0
17	1,087	44 (4.1)	0		8,759	63 (0.7)	0
18	1,118	48 (4.3)	0		9,852	43 (0.4)	0
19	1,146	62 (5.4)	0		8,682	68 (0.8)	0
20	1,238	65 (5.4)	0		9,621	55 (0.6)	0
21	1,197	55 (4.6)	0		10,248	64 (0.6)	0
22	1,121	48 (4.3)	0		11,264	83 (0.7)	0
23	1,166	41 (3.5)	0		9,546	82 (0.9)	0
24	1,041	37 (3.6)	0		9,962	81 (0.8)	0
25	1,075	42 (3.9)	0		8,910	96 (1.1)	0
26	1,032	35 (3.4)	0		7,735	68 (0.9)	0
27	976	31 (3.2)	0		7,431	80 (1.1)	0
28	922	28 (3.0)	0		7,567	83 (1.1)	0
29	945	26 (2.8)	0		7,306	80 (1.1)	0
30	908	28 (3.1)	0		6,998	82 (1.2)	0
31	887	20 (2.3)	0		7,824	76 (1.0)	0
32	911	17 (1.9)	0		7,484	74 (1.0)	0
33	848	19 (2.2)	0		7,176	82 (1.1)	0
34	925	11 (1.2)	0		8,018	85 (1.1)	0
35	883	16 (1.8)	0		8,186	84 (1.0)	0
36	856	11 (1.3)	0		8,768	85 (1.0)	0
37	833	13 (1.6)	0		9,549	86 (0.9)	0
38	821	12 (1.5)	0		8,788	82 (0.9)	0
39	773	14 (1.8)	0		7,217	73 (1.0)	0
40	844	17 (2.0)	0		6,448	68 (1.1)	0
41	755	12 (1.6)	0		5,513	63 (1.1)	0
42	721	10 (1.4)	0		5,284	79 (1.5)	0
43	733	16 (2.2)	0		5,746	73 (1.3)	0
44	766	18 (2.4)	0		6,599	70 (1.1)	0
45	803	26 (3.2)	0		5,655	75 (1.3)	0
46	846	28 (3.3)	0		4,234	83 (2.0)	0
47	815	42 (5.2)	0		5,054	82 (1.6)	0
48	859	40 (4.7)	0		4,683	95 (2.0)	0
49	935	46 (4.9)	0		5,493	101 (1.8)	0
50	947	51 (5.4)	0		5,488	112 (2.0)	0
51	1,004	43 (4.3)	0		4,151	117 (2.8)	0
52	1,066	52 (4.9)	0		4,840	119 (2.5)	0
2014							
01	1,124	62 (5.5)	0		6,497	113 (1.7)	0
02	1,091	70 (6.4)	1		7,313	121 (1.7)	0
03	1,198	79 (6.6)	3		6,401	126 (2.0)	0
04	1,257	91 (7.2)	0		6,089	118 (1.9)	0
05	1,286	86 (6.7)	1		6,048	105 (1.7)	1
06	845	32 (3.8)	1		4,656	68 (1.5)	1
07	1,036	86 (8.3)	5		4,172	98 (2.4)	1
08	1,122	70 (6.2)	4		4,340	113 (2.6)	0
09	1,123	77 (6.9)	1		6,850	111 (1.6)	0
10	1,084	63 (5.8)	1		5,925	117 (2.0)	0
Total	47,934	1923 (4.0)	16		349,712	4,149 (1.2)	3

*Real-time reverse transcription PCR testing for influenza A(H7N9) virus was implemented in April 1, 2013 (2013 week 14). Samples were collected from all patients with PUE and were laboratory tested. PUE, pneumonia of unknown etiology; ILI, influenza-like illness.

**Table 2:** Demographic, epidemiologic, and clinical characteristics of patients with severe and mild influenza A(H7N9) cases, Guangzhou, China, April 1, 2013–March 7, 2014*

Characteristic	Severe cases, n = 16	Mild cases, n = 4†
Sex ratio, M:F	11:5	1:3
Age, y, median (range)	66 (29–83)	5 (4–17)
Age group, y		
0–14	0	3 (75)
15–59	5 (31)	1 (25)
>60	11 (69)	0
Type of residence		
Urban	12 (75)	4 (100)
Rural	4 (25)	0
Occupation		
Retired	11 (69)	0
Housewife	2 (13)	0
Farmer	2 (13)	0
Tofu vendor in retail wet market	1 (6)	0
Kindergarten student	0	3 (75)
Primary or high school student	0	1 (25)
History of poultry exposure‡		
Direct contact	6§ (38)	0
Indirect contact	9¶ (56)	1# (25)
Unknown	3 (19)	3 (75)
Underlying medical conditions**	12 (75)	0
Preliminary diagnosis at the first hospital visit		
Influenza	0	3 (75)
Upper respiratory tract infection	6 (38)	1 (25)
Pneumonia	10†† (63)	0
Required hospitalization	16 (100)	1 (25)
Illness progressed to pneumonia	16 (100)	0
Received oseltamivir treatment	15 (94)	4 (100)
Admitted to intensive care unit	14 (88)	0
No. hospitals visited, median (IQR)	3 (1–4)	1 (1–2)
Time from illness onset to first medical care, median (IQR)	5 d (1–12 d)	6h (3–24h)
For patients in >60 y age group	7 d (2–12 d)	
Time from illness onset to antiviral therapy, median (IQR)	7 d (3–12 d)	1 d (6 h–4 d)
Time from illness onset to laboratory confirmation, median (IQR)	8 d (7–13 d)	3 d (2–5 d)
Length of illness, median (IQR)	24 d (11–32 d)	7 d (6–8 d)
Outcome		
Recovered and discharged	4 (25)	4 (100)
Still in hospital‡‡	1 (6)	0
Died	11 (69)	0

*Values are no. (%) patients except as indicated. LPM, live poultry market; IQR, interquartile range. †Includes the confirmed mild case detected from follow-up of close contacts. ‡Direct contact: bought poultry, slaughtered poultry, handled poultry meat, raised backyard poultry. Indirect contact: visited LPMs. §Four case-patients raised backyard chickens, 3 slaughtered live chickens, and 1 handled chicken meat. ¶Five case-patients visited LPMs daily; 4 visited LPMs several times. #Case-patient’s father managed a live poultry stall in a contaminated LPM; case-patient visited the stall several times. **Diabetes, hypertension, uremia, chronic obstructive pulmonary disease, and coronary heart disease. ††Nine case-patients were >60 y of age. ‡‡As of April 7, 2013.

During the same period (April 1, 2013–March 7, 2014), a total of 4,149 throat swab specimens were collected from 349,712 ILI patients (Table 1); 3 (0.1%) specimens were positive for influenza A(H7N9) virus. All 3 patients were young urban residents who had mild upper respiratory symptoms (Table 2). As a safety measure, these patients were isolated and treated with oseltamivir. All 3 patients recovered quickly (within 5–7 days) and were discharged after test results for throat swab samples were negative for 2 successive days.

During April–October 2013, 3 of 3,355 environmental samples collected from 24 LPMs were positive for influenza A(H7N9) virus, all on May 16. In contrast, of the 5,220 samples collected from 48 LPMs during November 2013 through March 7, 2014, a total of 141 (2.70%) samples were positive (Technical Appendix). A total of 375 poultry workers from 24 influenza A(H7N9) virus–contaminated LPMs were recruited and monitored, and 381 throat swab specimens were collected; repeat specimens were collected from 6 workers who showed symptoms. Asymptomatic influenza A(H7N9) virus infection was detected in 1 worker who managed a live poultry stall and had daily direct contact with live poultry. Two environmental samples collected from his stall on January 27, 2014, and a throat swab sample collected from the worker on January 28 were positive for influenza A(H7N9) virus. The worker was isolated, but test results for 3 consecutive throat swab specimens collected on January 30 and 31 and February 6 were negative, and in the absence of any symptoms or abnormal chest radiograph findings, he was discharged.

**Figure 2:**
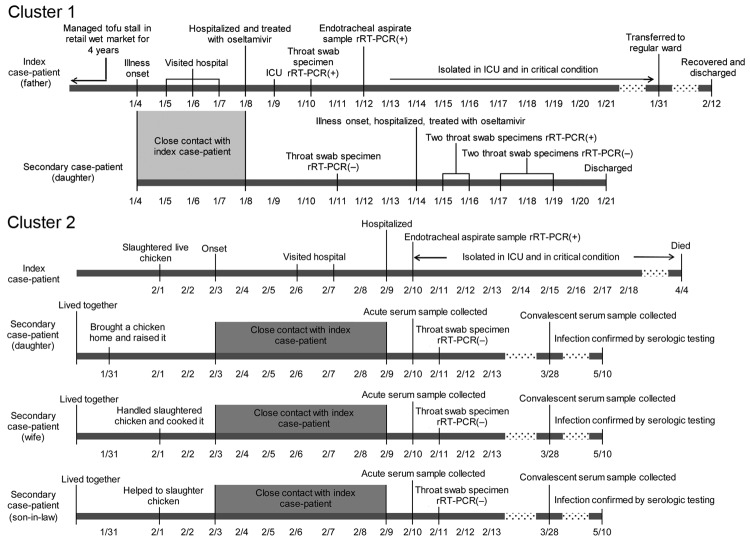
Timeline of illness for 2 family clusters of persons with confirmed influenza A(H7N9) virus infection, Guangzhou, China, 2014. ICU, intensive care unit; rRT-PCR, real-time reverse transcription PCR.

A total of 361 pairs of serum samples and 411 throat swab specimens were collected from 384 close contacts of influenza A(H7N9) case-patients; 2 family clusters were detected. In family cluster 1 (Figure 2), influenza A(H7N9) infection was laboratory confirmed in the index case-patient on January 10 and in 1 close contact (his daughter) on January 14 by positive test results on 2 throat swab specimens. The daughter showed mild respiratory symptoms and recovered quickly. She had no known history of poultry exposure before illness onset but had close, prolonged, and unprotected contact with her sick father. In family cluster 2, the index case-patient slaughtered a live chicken on February 1, became ill on February 3, and had influenza A(H7N9) infection laboratory confirmed on February 10. Three asymptomatic close family contacts of this patient had influenza A(H7N9) infection confirmed by a 4-fold rise in HI titer, although test results on throat swab specimens were negative. All 3 of these contacts had been involved in buying, slaughtering, or handling chickens and had close and unprotected contact with the index case-patient before he was isolated (Figure 2).

## Conclusions

Human infection with influenza A(H7N9) virus has been characterized by severe illness, in particular, rapidly progressive pneumonia and acute respiratory distress syndrome (*7*). However, the 21 case-patients with laboratory-confirmed influenza A(H7N9) that we identified in Guangzhou showed a wider spectrum of illness, ranging from severe pneumonia to mild ILI to asymptomatic infection. Clinical signs and symptoms differed notably across age groups; all mild cases occurred in those <20 years of age, whereas most severe cases occurred in older patients, similar to findings from previous studies (*8*,*9*). The age variances may be attributed to more frequent poultry exposure, more co-existing chronic diseases, or delayed medical admission and antiviral treatment among older patients.

Evidence shows the potential for influenza A(H7N9) virus transmission from person to person (*10**,**11*). In particular, epidemiologic findings of the father-daughter cluster indicate that person-to-person transmission may occur among family members after prolonged and intimate contact, consistent with findings in several other family clusters (*8**,**12**,**13*). However, no widespread mild influenza A(H7N9) infection was detected through ILI surveillance, which indicates that the likelihood of community-level transmission is low.

Subclinical influenza A(H7N9) virus infections of poultry workers have been identified by serologic testing (*14*). However, the possibility of cross-reactivity with other antigenically similar viruses cannot be ruled out. Using rRT-PCR, our surveillance identified a poultry worker with asymptomatic influenza A(H7N9) virus infection, providing further evidence for an occupational risk for asymptomatic infection.

Our study is limited by potential underreporting and by the increased use of PUE and ILI surveillance during the study period compared with previous periods. However, our results show that targeted surveillance during a period of elevated disease activity improved identification of mild or asymptomatic infections.

Technical Appendix Geographic distribution of confirmed influenza A(H7N9) cases and live poultry markets sampled in Guangzhou, China.
