# Supplementary material for: Asymptomatic, Mild, and Severe Influenza A(H7N9) Virus Infection in Humans, Guangzhou, China
Source: Emerg Infect Dis. 2014 Sep;20(9):1535–40. doi: 10.3201/eid2009.140424 (PMC4178418; doi:10.3201/eid2009.140424)
Supplement: Technical Appendix — Geographic distribution of confirmed influenza A(H7N9) cases and live poultry markets sampled in Guangzhou, China. [file 14-0424-Techapp-s1.pdf]

Article DOI: http://dx.doi.org/10.3201/eid2009.140424

Asymptomatic, Mild, and Severe Influenza A(H7N9) Virus Infection in Humans, Guangzhou, China

Technical Appendix

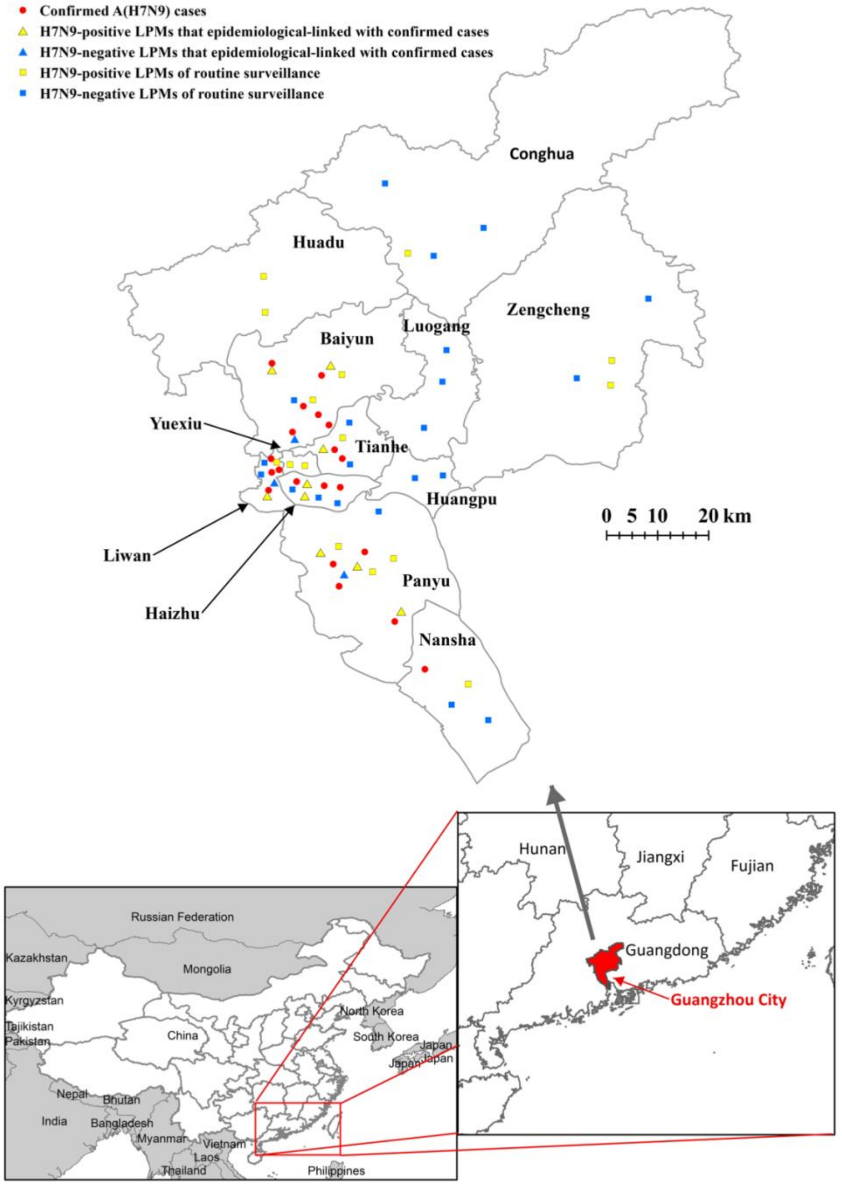

Technical Appendix Figure. Geographic distribution of confirmed influenza A(H7N9) cases and live poultry markets (LPMs) sampled in Guangzhou, China.
